# The Clinical and Prognostic Significance of Activated AKT-mTOR Pathway in Human Astrocytomas

**DOI:** 10.1155/2012/454957

**Published:** 2012-02-21

**Authors:** Elias A. El Habr, Christos Adamopoulos, Georgia Levidou, Aggeliki A. Saetta, Penelope Korkolopoulou, Christina Piperi

**Affiliations:** ^1^First Department of Pathology, Laiko Hospital, National and Kapodistrian University of Athens Medical School, 11527 Athens, Greece; ^2^Department of Biological Chemistry, National and Kapodistrian University of Athens Medical School, 11527 Athens, Greece

## Abstract

Astrocytomas, the most common type of gliomas, and especially grade IV glioblastomas are “endowed” with strong proliferation and invasion potentials, high recurrence rate, and poor patients' prognosis. Aberrant signaling of AKT-mTOR (mammalian target of rapamycin) has been implicated in carcinogenesis. This paper is focused on the impact of deregulated AKT-mTOR signaling components in the clinical outcome and prognosis of human astrocytomas. Current therapeutic targeting of astrocytomas with AKT-mTOR inhibitors in preclinical and clinical stage is also discussed, including future perspectives regarding the management of these devastating tumors.

## 1. Introduction

Gliomas present the commonest type of tumor of the central nervous system. Based on the World Health Organization (WHO) criteria, diffuse astrocytomas, the most aggressive type of gliomas, are further classified into varying degrees of malignancy ranging from grade II to IV [1]. The life expectancy of patients diagnosed with a grade IV astrocytoma is on average 14 months after diagnosis despite differential treatment strategies including surgery, radiation, and/or chemotherapy.

The development of phosphorylation state-specific antibodies (PSSAs) has enabled a static determination of protein phosphorylation in the spatially complex structures of cells and tissues [2]. What is expected from PSSAs is the ability to confer a “screenshot” of intracellular signal transduction pathways, so as to provide important information to the clinician regarding prognosis, prediction, and/or therapy [2].

It is well established that the deregulation of AKT-mTOR (mammalian target of rapamycin) signaling is involved in carcinogenesis and plays a major role in the development of an aggressive phenotype influencing prognosis and determining response to therapies. The aim of this paper is to critically discuss and compose the results of several reports dealing with the role of activated AKT-mTOR pathway in human diffuse astrocytomas. We will focus on reports that used PSSAs for the activated form of AKT, mTOR, p70S6K, S6, and 4E-BP1 and highlight the role of these molecules in gliomagenesis.

## 2. The AKT-mTOR Pathway

### 2.1. A Quick Overview (Figure 1)

AKT is one of the most important downstream targets of PI3K (phosphoinositide 3′-kinase). The AKT-mTOR pathway is initially activated at the level of cell membrane, and propagation of the activation signal occurs through PI3K class IA. A variety of signaling molecules including tyrosine kinase growth factor receptors (e.g., epidermal growth factor receptor (EGFR), insulin-like growth factor-1 receptor (IGF-1R)) as well as G-protein-coupled receptors, cell adhesion molecules, and oncogenes may lead to PI3K activation [3]. Phosphatidylinositol-3,4,5-triphosphate (PIP3), PI3K product, binds to 3′-phosphoinositide-dependent kinase 1 (PDK-1) and AKT through the pleckstrin homology domains (PH). This allows the translocation of both proteins to the cell membrane, followed by their activation [4]. Colocalization of AKT with PDK1 results in phosphorylation of AKT at Thr308 and its partial activation [5]. In order for AKT to be fully activated, an additional Ser473 phosphorylation by the putative kinase PDK2 is required, which is believed to be mTORC2 (mTOR complex 2) [6]. PI3K is antagonized by PTEN (phosphatase and tensin homolog deleted on chromosome 10) through dephosphorylation of PIP3, thereby preventing AKT translocation and subsequent activation of both AKT and PDK-1. Once activated, AKT moves to the cytoplasm and nucleus, where it phosphorylates, activates, or inhibits many downstream targets to regulate various cellular functions including cell metabolism, protein synthesis, cell survival/inhibition of apoptosis, and cell cycle progression.

mTOR, a serine/threonine kinase, presents AKT's most well-studied downstream substrate. mTOR can be either directly phosphorylated and activated by AKT or indirectly activated through phosphorylation and subsequent inactivation of TSC2 (tuberous sclerosis complex 2, known as tuberin). TSC2 usually inhibits mTOR via Rheb (Ras homolog enriched in brain), a GTP-binding protein. Phosphorylation of TSC2 leading to its inactivation allows Rheb kept in the GTP-bound state, further inducing increased mTOR activation [3]. Two complexes of mTOR exist including the complex of mTORC1, with mTOR binding to Raptor, and the complex mTORC2, with mTOR binding to Rictor. Among several functions of mTOR, the regulation of translation is the best studied in relation to oncogenesis [7]. Two downstream pathways of mTORC1 involved in the translation machinery are responsible for ribosome recruitment to mRNA: phosphorylation and inactivation of 4E-BP1 (eukaryotic translation initiation factor 4E-binding protein 1), the repressor of mRNA translation, and activation of S6K1 (ribosomal S6 kinase 1), the promoter of mRNA translation [8]. 4E-BP1 phosphorylation (p-4E-BP1) prevents eIF4E interaction, liberating it to interact with eIF4G to form the eIF4F complex that enhances the cell's overall translational machinery [9]. Because eIF4E is the least abundant among the initiation factors, its dissociation from 4E-BPs represents the rate-limiting event for cap-dependent initiation of translation. Many growth- and proliferation-related proteins encoded by “weak” mRNAs show great reliance on the availability of eIF4E [10]. S6K1, which is phosphorylated and activated by mTORC1 on a hydrophobic motif, further phosphorylates S6, the 40S ribosomal protein, enhancing mRNAs translation with a 5′-TOP (5′-terminal oligopolypyrimidine). All ribosomal proteins and elongation factors are encoded by 5′-TOP [11].

### 2.2. AKT Protein: A Basic “AKTor” in Human Astrocytomas

AKT (also known as protein kinase B, PKB) is a serine/threonine kinase with three isoforms being identified in mammalian cells: AKT1 (PKB*α*), AKT2 (PKB*β*), and AKT3 (PKB*γ*). They are all encoded by different genes with ubiquitous expression in normal cells and variable levels of expression among tissues [27]. AKT is a central node of PI3K signaling pathway, with crosstalk and feedback loops influencing its regulation. Altered expression of AKT has been associated with carcinogenesis [28].

Several studies [12–26] have investigated the clinical and prognostic significance of AKT and its activated form (phosphorylated AKT, p-AKT) in human astrocytomas (Table 1), without discrimination between the nuclear and cytoplasmic localization of this molecule apart of a few reports. The subcellular localization of p-AKT is, however, very important since current data indicate differentially shaped signaling in a spatially restricted way [13]. Interestingly, increased levels of nuclear p-AKT expression were correlated with higher levels of cytoplasmic immunostaining [13, 15]. These correlations between nuclear and cytoplasmic immunoexpression, along with the concurrent nuclear and cytoplasmic localization of p-AKT, are compatible with the dual regulatory role of gene expression targeting proteins in both cytoplasmic and nuclear level. It is worthy of note the findings of the study of Annovazzi et al. [18] showing that the immunostaining of p-AKT was nuclear in low-grade gliomas and cytoplasmic in high-grade gliomas. The reason for this disparity is unclear despite the explanation given by the latter group supporting that the prevailing nuclear localization in low-grade gliomas, where AKT expression is infrequent, could mean that the regulatory mechanism is different in comparison with tumors with cytoplasmic localization.

All reports dealing with different grades of human astrocytomas, except one, showed that increased total, nuclear, and/or cytoplasmic AKT and p-AKT correlated with tumor histological grade (Table 1) indicating that activation of AKT participates in astrocytic tumor progression. On the contrary, Hlobilkova et al. [20] found the same proportions of p-AKT expression between low- and high-grade astrocytomas, suggesting that its activation is a starting event in gliomagenesis. Yang et al. [12] showed a significant correlation of p-AKT expression with KPS (Karnofsky Performance Status) score, whereas a significant association between cytoplasmic p-AKT expression and patients' age was denoted by Saetta et al. [13]. Also, from the same study [13], correlations of nuclear p-AKT expression with VEGF (vascular endothelial growth factor) and microvessel density were observed, which implicate p-AKT as major component of angiogenesis in astrocytic gliomas. Matsutani et al. [19] found that patients positive for AKT with residual tumors postoperatively exhibited tumor recurrences, and invasive recurrence into surrounding brain was present only in tumors overexpressing AKT.

Two independent studies [13, 22], unlike Mizoguchi et al. [21] and Choe et al. [26], observed that p-AKT expression correlates to expression of p-ERK in gliomas. This correlation can be explained by the simultaneous activation of these parallel pathways by common receptor tyrosine kinases (e.g., EGFR) acting upstream. This can be further supported by the correlation of p-AKT activation with EGFR gene amplification and with progression from anaplastic astrocytomas to glioblastoma [18, 21]. More specifically, the immunostaining of wild type-EGFR (EGFRwt) [18, 21] and mutant EGFR (EGFRvIII) [21] correlated with activation of AKT. Also, from the latter report [21], a strong association between p-AKT and expression of p-STAT3 was denoted, whereas the statistical analysis of two independent studies [23, 24] failed to confirm such a relation. On the other hand, the immunohistochemical data from the microarray study of Wang et al. [24] showed an important correlation between p-AKT and activated NF-*κ*B, suggesting that AKT activation may lead to NF-*κ*B activation in diffuse astrocytomas. The finding of Chakravarti et al. [25] that shows an inverse association of p-AKT levels with cleaved caspase 3 (an apoptotic marker) levels indicates that PI3K members may act towards suppression of apoptosis, suggesting a possible explanatory mechanism by which the PI3K pathway may enhance resistance to radiation therapy in grade IV astrocytomas. Such a finding did not emerge from the study of Annovazzi et al. [18] probably due to technical reasons.

Since the expression status of mutant *IDH1* (isocitrate dehydrogenase 1) has been recently proposed as a prognostic factor in astrocytic tumors [29], Saetta et al. [13] included the IDH1-R132H antibody in their immunohistochemical analysis, which is a surrogate marker of the *IDH1* mutant, in a subset of cases in order to evaluate any potential relationship with p-AKT, but the expression of the former was not related neither to nuclear nor to cytoplasmic p-AKT. Also, the same group in a latter study [30] (see below) examined any possible correlation of IDH1-R132H expression with the activated forms of mTOR, p70S6K, and 4E-BP1. All three proteins were found to be unrelated to IDH1-R132H expression.

The relationships of p-AKT with p-mTOR and its downstream molecules will be discussed in next sections. From some reports, several correlations of p-AKT and its upstream regulators have emerged. *In vitro* experiments have shown that addition of YKL-40 protein to cells of connective tissue resulted in increased cell proliferation through AKT activation and MAPK pathways, nominating *YKL-40* as a potential regulator of these signaling pathways, since its secreted form is the product of one of the most expressed genes in glioblastoma with prognostic potential for these tumors [22]. YKL-40 expression was marginally correlated with expression of p-AKT in the study of Pelloski et al. [22], but, from the same study, no correlation was observed among expression of p-AKT and PTEN. On the contrary, Choe et al. [26] found a strong inverse correlation between AKT activation and PTEN loss, which remained significant during multivariate analysis. Also, from the same report [26], a statistical correlation between p-AKT and p-FKHR emerged and was maintained as an independent association during multivariate analysis. Since AKT is an important mediator of PI3K, a significant association among the activation states of these two proteins was expected [25].

The prognostic importance of AKT or p-AKT in astrocytic tumors is controversial based on current literature. The study of Matsutani et al. [19] found overexpression of AKT to be significantly associated with progression-free survival (PFS) and shorter overall survival (OS). In other reports, p-AKT expression was predictive of worse prognosis [12, 17, 21, 25]. Chakravarti et al. [25] showed elevated expression levels of p-AKT to be significantly associated with reduced time to death in the entire cohort, but such a relation did not remain significant during multivariate survival analysis. They then examined the prognostic significance of p-AKT only in patients diagnosed with a grade IV astrocytoma treated by surgery combined with postoperative radiation and showed a correlation of p-AKT expression with adverse outcome, suggesting an important role of this pathway in radiation resistance. Yang et al. [12] found also that p-AKT expression was statistically associated with worse prognosis, but, unlike Chakravarti et al. [25], in multivariate survival analysis, p-AKT emerged as an independent factor of prognosis. In the same context, the report of Suzuki et al. [17] showed that rate of survival for p-AKT positive tumor patients was lower than that of p-AKT negative tumors, whereas multivariate survival analysis nominated p-AKT as a strong independent prognostic factor. During univariate survival analysis of Saetta et al. [13], p-AKT expression did not correlate with survival, whereas multivariate analysis revealed cytoplasmic p-AKT expression as an independent prognostic factor, implying a higher survival probability. The latter finding is not one of a kind, since it is in accordance with current observations in lung and ovarian tumors [31, 32]. Finally, in two independent reports [18, 22], p-AKT expression failed to attain prognostic significance in gliomas.

### 2.3. The Role of mTOR Protein in Astrocytomas

The mTOR proteins belong to the PIKK (phosphoinositide 3-kinase-related kinase) family, transmitting signals associated with cellular growth, proliferation, and stress responses [33]. mTOR is a large protein of ~300 kDa Mwt with a COOH-terminal catalytic domain homologous to PI3K, functioning exclusively as a protein serine/threonine kinase [34].

Some reports [14, 18, 22, 23, 26, 30] have investigated the clinical and prognostic significance of activated mTOR (phosphorylated mTOR, p-mTOR) in human astrocytomas.  Table 2 summarizes the important findings of these reports. In the report of Korkolopoulou et al. [30], mTOR was seen predominantly in the cytoplasm/cell membrane but also occasionally in the nucleus, suggesting that this nuclear import of mTOR may have an important role in activating its cytoplasmic signaling. On the other hand, Li et al. [14] found that the immunostaining of p-mTOR was both nuclear and cytoplasmic, whereas Annovazzi et al. [18] observed a nuclear immunostaining. The reason for the disparity in mTOR immunostaining may be due to the difference in the antibodies used and requires further investigation.

It is worthy of note that 3 independent reports [14, 18, 30] dealing with different grade of astrocytomas denoted a correlation of p-mTOR expression with tumor grade. These findings imply that activation of mTOR may be a late event during gliomagenesis, facilitating the acquisition of a malignant phenotype.

An association between VEGF and cytoplasmic p-AKT levels in astrocytic tumors was discussed previously in Section 2.2 [13]. The same group [30] denoted that this relationship also applies to p-mTOR and its target proteins p-4E-BP1 and p-p70S6K, which was illustrated in their similar distribution in perinecrotic areas of glioblastomas (Tables 3 and 4). Their findings are supported by recent data clearly placing HIF-1a the driving force of VEGF upregulation and angiogenesis under hypoxic conditions downstream of mTOR [37], as well as from *in vitro* experiments where decreased VEGF levels with suppression of angiogenic phenotype followed mTOR inhibition or enhanced translation of VEGF mRNA followed hypoxic activation of 4E-BP1 and eIF4E [30]; Beclin 1, expression of which decreases with malignancy, was inversely correlated with p-mTOR [18].

Since AKT and mTOR proteins interact with and activate each other as discussed in Section 2.1, it is expected the activated forms of these two proteins to be correlated together. Indeed, several reports have denoted a correlation of these two proteins [18, 22, 26, 30]. Choe et al. [26] found a strong correlation of p-AKT and p-mTOR in univariate analysis, but, in multivariate, this association did not reach significance, concluding that additional inputs (e.g., nutrients, amino acids, cellular ATP, and phosphatidic acid) to mTOR activation are involved, some of which may be associated with other parts of the signaling pathway. Only one report [23] did not observe association between p-AKT and p-mTOR suggesting that multiple AKT-independent signaling pathways on mTOR regulation could be involved. Pelloski et al. [22], unlike Korkolopoulou et al. [30], showed a strong p-mTOR correlation with p-ERK, which could be explained by the simultaneous stimulation of these parallel pathways by EGFR, the expression of which was correlated with both proteins in the analysis of Choe et al. [26]. Pelloski et al. [22], also, found a correlation of p-mTOR with YKL-40 (see Section 2.2) and PTEN expression. The latter finding is in accordance with previous data of a relationship between high PTEN expression and AKT activation [22]. The contradictory findings in the literature suggest a tumor specific relationship between PTEN expression and activation of the AKT pathway and this should be better assessed by alternative methodology than immunohistochemical analysis.

As mentioned above, the most important effectors of mTOR kinase in the regulation of translation are 4E-BP1 and p70S6K, S6 ribosomal protein, being the major effector of the latter. In this context, Choe et al. [26] found an association between p-mTOR and p-S6, whereas Pelloski et al. [22] denoted a strong association of the former protein with p-p70S6K. Korkolopoulou et al. [30] found also correlations of p-mTOR with p-4E-BP1 and p-p70S6K, the former of marginal significance, but, despite these interrelationships, the three proteins were topographically distinct, since only p-mTOR was found in pseudopalisading perinecrotic cells in glioblastomas. This finding is one of a kind and could attribute a functional link with tumor hypoxia, confirmed by *in vitro* data indicating upregulation of mTOR by hypoxia inducible factor-1a (HIF-1a) [37].

Regarding the prognostic significance of activated mTOR, few reports [18, 22, 30] have investigated the influence of p-mTOR on patients' survival. From the survival analysis of Annovazzi et al. [18], no significant correlations of p-mTOR with patients' survival emerged. On the contrary, Pelloski et al. [22] and Korkolopoulou et al. [30] found that p-mTOR expression was correlated with short OS and free of disease survival, respectively, but such a relation did not remain significant during multivariate survival analysis.

### 2.4. Implication of p70S6K and Its Major Effector S6 Protein in Astrocytomas

There are two isoforms of S6K1 (cytoplasmic p70S6K1 and nuclear p85S6K1) present in mammals, produced by alternative splicing from the same transcript [38]. The large isoform, p85S6K1, is composed of an N-terminal 23-aa long, which directs p85S6K1 to the nucleus. On the contrary, the short form, p70S6K1, is mainly located in the cytoplasm. Current data indicate a similar regulation of p85S6K1 and p70S6K1. Initial mapping of the main residues for p70S6K1 activation was to T229 (of activation loop) and T389 (of the hydrophobic motif) and subsequently to S371 of the linker domain [39].

Table 3 shows the reports dealing with the significance of p70S6K in human astrocytomas. p70S6K is a nuclear cytoplasmic shuttling protein, activated in the nucleus by mTOR and relocalized in the cytoplasm after mRNA translation initiation [40]. Thus, it is not surprising that p-p70S6K has been reportedly seen in the cytoplasm and/or in the nucleus [14, 30]. Two independent reports [14, 25] denoted a correlation of p-p70S6K with tumor grade suggesting that the activation of this protein takes place later during gliomagenesis. On the contrary, Korkolopoulou et al. [30] denoted that p70S6K activation may take place at an earlier stage of the neoplastic process, since no difference existed between low- and high-grade cases.

The latter group [30] observed a correlation of cytoplasmic p-AKT with p-p70S6K. Interestingly, in multidimensional analysis in glioblastomas, p-p70S6K, but not with p-mTOR, clustered together with p-AKT implying that the downstream effect of p-AKT is primarily conveyed by S6K signaling through TSC2 [23]. The same correlation between p-AKT and p-p70S6K was denoted by 3 different groups [22, 23, 25]. Since p70S6K is an important downstream mediator of PI3K, a significant correlation among the activation states of both proteins is expected and further predicted by preclinical models. In accordance, p-PI3K levels and p-p70S6K were correlated in the study by Chakravarti et al. [25]. Regarding YKL-40 as discussed in Section 2.2 and 2.3, its expression correlated significantly with p-p70S6K [22]. The same report denoted a strong correlation of the latter with p-ERK; since the activation of AKT was inversely associated with cleaved caspase 3, it was expected that the same correlation will occur between p-p70S6K and the latter, which was found by Chakravarti et al. [25], suggesting an implication of activated p70S6K in the suppression of apoptosis.

Pelloski et al. [22] found that p-p70S6K was associated with reduced overall survival time on univariate but not in multivariate analysis. A previous study [25] showed this marker to be significantly correlated to survival on both analysis types. This can be explained statistically since the percentage of p-p70S6K-negative cases in the former study (6%) was lower than that in the latter study (44%) or because Pelloski et al. [22] included, in their study, additional markers (namely, YKL-40 and p-ERK) with more dominant molecular effects within tumor cells. Finally, the survival analysis of Korkolopoulou et al. [30] did not reveal any association of p-p70S6K with patients' survival.

The 40S ribosomal subunit protein S6 is the first identified and the most well-studied substrate of S6K1. Its phosphorylation has been found to correlate with increased protein synthesis. After stimulation, S6K1 phosphorylates S6 on five serine residues (S235, S236, S240, S244, and S247) within the C-terminus [38].

Table 3 summarizes the findings of several reports discussing the role of activated S6 (phosphorylated S6, p-S6) in human astrocytomas. Yang et al. [12] denoted a cytoplasmic immunostaining for p-S6, whereas Annovazzi et al. [18] found that grade II had different immunostaining for p-S6 than grade III and IV (nuclear for grade II and III, and cytoplasmic for grade IV). Similar finding emerged for p-AKT in the same report and was discussed in Section 2.2. Also, noteworthy is the findings of McBride et al. [35] who used two different antibodies for the activated form of S6 (Table 3). Both antibodies showed the same percentage of immunostaining, and their expressions were strongly correlated, which served as a good internal validation for their IHC (immunohistochemistry) protocol. Also, Annovazzi et al. [18] validated their IHC by WB (Western blot) analysis and found a strong correlation between the findings of both techniques; a significant correlation of p-S6 expression with tumor grade was denoted by 2 different groups [12, 18], whereas Ermoian et al. [36] did not find such a relation; lower KPS was correlated with p-S6 [12].

A protein-positioned downstream of PTEN, namely, PRAS40, does not inhibit the mTOR pathway in its phosphorylated form (discussed in Section 2.1), and it can therefore be assumed that upon activation of the mTOR pathway, these proteins are linked and correlate in terms of phosphorylation status. In accordance, McBride et al. [35] showed association of S6 and PRAS40 phosphorylation along with *PTEN* methylation. In addition, S6 phosphorylation and PTEN expression were negatively correlated. In the same context, Riemenschneider et al. [23] found a strong correlation between p-AKT and p-S6. Finally, Annovazzi et al. [18] found a strong positive correlation of p-S6 with Ki67 and negative with Beclin 1 (see Section 2.3).

McBride et al. [35] performed two different survival analyses for their cohort, one with the anti-p-S6 antibody for the 235/236 epitope and one for the epitope 240/244. In both cases, an inverse correlation between OS and phosphorylation of S6 was denoted. In the same context, Yang et al. [12] found a strong correlation between p-S6 expression and worse prognosis, which in multivariate survival analysis emerged as an independent prognostic factor. On the contrary, the survival analysis of Annovazzi et al. [18] and Ermoian et al. [36] did not reveal any correlation of p-S6 with patients' survival.

### 2.5. 4E-BP1 Protein: The Less Studied in the Pathogenesis of Astrocytomas

4E-BP1 (also known as PHAS-1) is a translation repressor protein and one of the main effectors of mTOR in the PI3K/AKT signaling pathway that integrates signals from extracellular stimuli, amino acid availability, and oxygen and energy status of the cells. 4E-BPs contain a TOS (TOR signaling) motif that binds the mTORC1, which controls the activity of 4E-BP1 [41]. mTORC1 activation leads to 4E-BP1 phosphorylation at Thr36, Thr45, Ser64, and Thr69 sites and 4E-BP1 release from eIF4E [41].

Table 4 summarizes the important findings of the few reports dealing with the role of activated 4E-BP1 (p-4E-BP1) in human astrocytomas. In the report of Korkolopoulou et al. [30], the immunostaining of p-4E-BP1 was nuclear (nuclear cytoplasmic shuttling protein). Although the mechanisms of the nuclear localization of p-4E-BP1 have not been clarified, there is current evidence that eIF4E functions as a nuclear regulator of several RNAs exportation implicated in proliferation and cell growth [42]. Also, Korkolopoulou et al. [30] showed that p-4E-BP1 expression levels increased with grade, whereas, in the report of Ermoian et al. [36], no correlation was elicited between p-4E-BP1 mRNA or protein levels (as measured using immunoblotting) and glioma grade.

p-4E-BP1 expression correlated with cytoplasmic p-ERK expression in the report of Korkolopoulou et al. [30], consistent with *in vitro* data indicating the emerging role of ERK signaling in regulating 4E-BP1 [43]. Also, from the latter report [30], a significant correlation of p-4E-BP1 expression with nuclear p-AKT was denoted, which was in accordance with the data of Ermoian et al. [36] that observed a similar association; an important finding emerging from these two reports is the absence of correlation between p-4E-BP1 and p-S6 [36], from on one hand, and among p-4E-BP1 and p-p70S6K, on the other hand [30]. This finding shows that, in the regulation and activation of these two molecules, other pathways may be implicated; VEGF, which is a marker of angiogenesis (see previous sections), was also found to be correlated with p-4E-BP1 [30].

One of the most important findings emerging from the investigation of Korkolopoulou et al. [30] is the independent adverse prognostic significance of p-4E-BP1 expression, in the entire cohort and glioblastomas, which has not been documented thus far. *In vitro* experiments have documented sensitization of U87 glioblastoma xenografts to irradiation following 4E-BP1 targeting and decreased hypoxia tolerance, indicating that glioblastomas, which are known to be hypoxic, are expected to rely more on translation regulation pathways for critical functions than their lower grade counterparts [44] thus corroborating the finding of Korkolopoulou et al. [30] that the prognostic effect of 4E-BP1 was established in glioblastomas.

### 2.6. Therapeutic Targeting of AKT-mTOR Pathway in Astrocytomas

**Figure 1:**
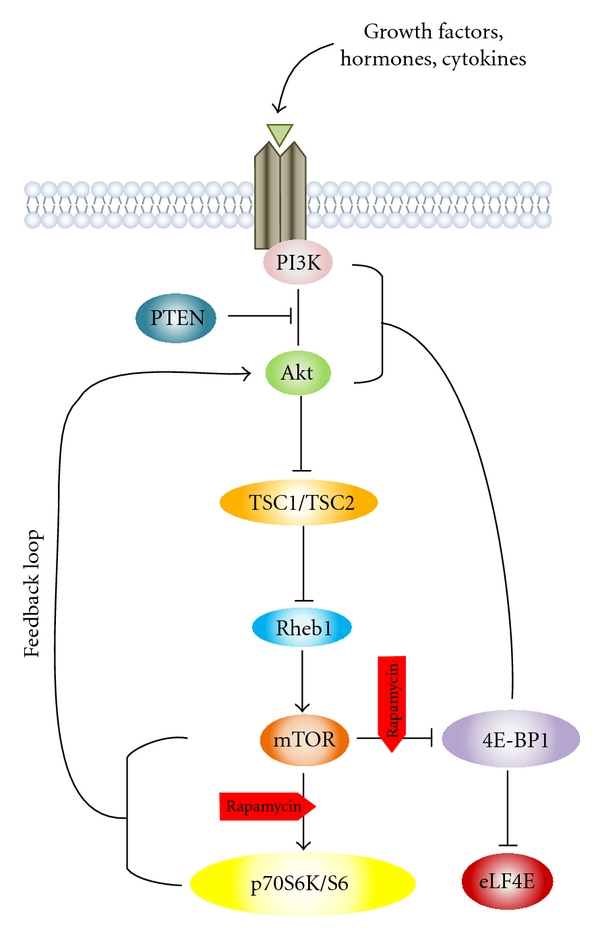
Schematic representation of AKT-mTOR signaling pathway showing rapamycin inhibition sites in astrocytomas.

The development of small kinase inhibitors has improved clinical practice for several solid tumors and presented a reasonable strategy for astrocytomas treatment. Primary glioma cell cultures as well as U87 and U251 cell lines where a strong activation of AKT/mTOR pathway was observed exhibited increased sensitivity to rapamycin, the archetypic mTOR inhibitor which did not, however, caused a compensatory AKT activation resulting from mTORC1 negative feedback via insulin receptor substrate (IRS1, Figure 1) [30, 45]. Additionally, rapamycin was effective in inhibiting the phosphorylation of both p70S6K and 4E-BP1 even at 48 hours [30] being a clinical promise in recurrent glioblastoma patients displaying higher levels of p-p70S6K in baseline tumor samples [46].

Unfortunately, single agent mTOR inhibitors such as rapamycin (sirolimus, CCL-779) that specifically complexes with FK506-binding protein 12 (FKBP12), thereby interacting allosterically and inhibiting mTOR (Figure 1), has produced minimal clinical activity and improvement in neuroimaging of recurrent glioblastoma patients [47, 48]. The PFS6 (progression-free survival at 6 months) was rather low at 7.8%, whereas the median OS was 4.4 months. Additional, the rapalogs, temsirolimus (CCI-779), deforolimus (AP23573), and everolimus (RAD001) have also been generated without major clinical benefit, and, although they are all well tolerated, some toxicities have been reported including lymphopenia, hyperlipidemia, stomatitis, and increased risk of opportunistic infections [46, 48, 49]. Furthermore, a rapamycin phase I trial was conducted in recurrent glioma patients deficient in PTEN based on preclinical data where PTEN mutant/null tumors exhibited an enhanced susceptibility to mTOR inhibition [50–52]. Interestingly, a complete inhibition of proliferation was observed in half of the patients which correlated to mTORC1 inhibition.

Taking into account the complexity of intracellular signal transduction pathways and the ability of tumor cells to compensate for acute changes, it is not surprising that single-agent therapies have a limited efficacy against most solid tumors, including gliomas [45]. The possibility of an upstream positive feedback loop at the PI3K level has driven therapeutic targeting towards multiple signaling molecules of the EGFR-PI3K-mTOR axis and identification of agents showing additive or synergistic antitumor effects [28]. Indeed, preclinical studies of combined EGFR and mTOR inhibitors have shown antiproliferative and proapoptotic effects against gliomas [45, 53]. In accordance, several pilot studies with drug combinations in recurrent gliomas showed either a promising PFS6 at 25% for glioblastoma patients [54] or disease stabilization [55].

Initial phase I studies indicated that combination treatment in recurrent glioblastoma patients was well tolerated resulting either in stabilization of the disease for some patients or partial response rates [54, 56]. However, in a more recent phase I and II study of recurrent glioblastoma, combined treatment use of erlotinib and temsirolimus caused increased toxicity without major clinical advantages. Temsirolimus was maximally tolerated at 15 mg per week (lower dose than used in monotherapy) and erlotinib at 150 mg per day. Most common grade 3 toxicity was rash followed by severe mucositis, thrombocytopenia, and diarrhea. On phase II trial, PFS6 of 40 glioblastoma patients was 12.5% with the median PFS being 8 weeks and only 30% of patients exhibiting stable disease. Trials involving combinations of cytotoxic drugs with mTOR inhibitors have been initiated recently aiming in reducing the immunosuppressive consequences. 

Several factors may contribute to this minimal antitumor response to combination therapy of EGFR and mTOR inhibitors in glioblastomas, including mainly therapy resistance or use of inadequate drug doses [57]. It is evident that glioblastomas exhibit molecular heterogeneity involving EGFRvIII and PTEN mutations, increased AKT activation, or MGMT promoter methylation that may differentiate the efficiency of EGFR-AKT-mTOR cascade targeting among patients. Although several studies have been performed aiming to establish molecular profiles that would predict predictive clinical responses towards EGFR and/or mTOR inhibition, a convincing molecular signature for glioblastoma is still elusive. Additional to identification of driver mutations as therapeutic targets, efficient target inhibition is also important. The use of rapamycin and its derivatives as potent mTOR inhibitors at the current clinical doses has been challenged recently, especially regarding inhibition of 4E-BP1 and proliferation in some patients [58]. A better activity towards this pathway is expected from next generation mTOR inhibitors [58, 59]. It has been argued that modulation of 4E-BP1 function may serve to determine the sensitivity of tumor cells to PI3K/AKT pathway inhibitors. It is thus conceivable that 4E-BP1 modulation may have a place in the treatment options for glioblastoma as an adjunct to the existing or upcoming cytotoxic therapies.

## 3. Conclusion

It is evident that AKT-mTOR pathway activation is highly implicated in glioma biology contributing to tumor progression and angiogenesis. Many reports, however, analyzing the same molecule in gliomas had often controversial or discrepant findings, preventing us to generate reliable conclusions regarding the clinical and prognostic importance of these molecules in astrocytomas. This discrepancy could be attributed to differences in techniques used, antibodies, cohorts, and so forth. What was missing from most reports cited in this paper was the validation of their findings. For example, in the study of Korkolopoulou et al. [30], the IHC was validated by WB and by Rapamycin treatment, whereas McBride et al. [35] used two different antibodies for the same protein (p-S6), which served as an internal validation for their IHC. On the other hand, Pelloski et al. [22] validated the findings of their IHC by using a validation cohort different from their main cohort enrolled in the study.

Despite these remarks, the implication of activated AKT-mTOR pathway in the pathogenesis of human astrocytomas could not be ignored. Some findings of the cited reports were similar and allowed us to generate a safe conclusion. For example, activated AKT was seen to correlate with tumor grade in all cited reports except one. More studies are needed in order to validate the findings of the cited reports, especially for mTOR and the effectors 4E-BP1 and p70S6K.

Furthermore, the AKT-mTOR pathway remains a promising and potential target for glioblastoma therapy. Due to the molecular heterogeneity of glioblastomas, it is evident that strategies targeting this pathway require considerable advances in genetics and pharmacology. Genomic approaches of large scale are required for identification of main driver mutations and establishment of molecular signatures leading to successful application of mTOR-targeted therapy. Screening tumor samples for compensatory receptor tyrosine kinases will identify those patients that require combinations of multiple kinase inhibitors for successful downstream signaling suppression [60]. The recent categorization of glioblastoma subtypes is an important tool in identifying tumors that are more susceptible to mTOR targeting [61].

Radiotherapy in combination with mTOR inhibition (temsirolimus) is an alternative schedule that has proved efficient in preclinical studies [62] and is currently under evaluation in a clinical study of newly diagnosed glioblastomas in comparison to chemoirradiation and lack of MGMT methylation [63].

Finally, advances in pharmacological approaches have resulted in production of novel mTOR inhibitors with an expected superior activity than rapalogs [59, 64]. Interesting clinical data are expected from a current phase I trial where combination of mTORC1 and 2 inhibitors as well as combination of mTORC1 and 2 and PI3K inhibitors has been applied to patients with glioblastomas that have undergone radiation therapy.

## Figures and Tables

**Table 1:** Summary of reports investigating the role of AKT and p-AKT in astrocytomas.

Report (number of cases and grades)	Antibody used	Immunostaining percentage	Correlations with clinicopathological features	Other correlations	Survival analysis
Yang et al. [12] (96 patients: 16 grade II, 35 III, and 45 IV)	Rabbit monoclonal anti-p-AKT (Ser473) ab (CST), at a concentration of 1.5 Ig/mL (IHC)	92.7% (89/96) showed nuclear and cytoplasmic staining	p-AKT with (i) higher grade (ii) lower KPS score	—	(i) p-AKT associated with a worse prognosis (ii) Multivariate analysis: p-AKT as an independent prognostic factor
Saetta et al. [13] (82 patients: 20 grade II, 14 III, and 48 IV)	Rabbit polyclonal anti-p-AKT1/2/3 ab (SCB), diluted 1 : 250 (IHC)	p-AKT: (i) nuclear 93.05% (67/72) (ii) cytoplasmic 59.72% (43/72)	(i) Nuclear and cytoplasmic p-AKT with tumor grade (ii) Cytoplasmic p-AKT with patients' age	(i) Nuclear p-AKT with cytoplasmic p-AKT (ii) Nuclear and cytoplasmic p-AKT with nuclear and cytoplasmic p-ERK (iii) Nuclear p-AKT with VEGF and MVD	Multivariate analysis: cytoplasmic p-AKT as independent predictor of survival (higher survival probability)
Li et al. [14] (87 patients: 27 grade I-II, 24 III, and 36 IV)	Rabbit monoclonal anti-p-AKT ab (EP), diluted 1 : 200 (IHC)	(i) 72.4% (63/87) showed nuclear and/or cytoplasmic staining (ii) 36.1% of grade IV showed strong expression	High p-AKT levels with tumor grade	—	—
El-Habr et al. [15] (71 patients: 7 grade II, 5 III, and 59 IV)	Rabbit polyclonal anti-p-AKT1/2/3 ab (SCB), diluted 1 : 250 (IHC)	p-AKT: (i) nuclear 97% (22/24) (i) cytoplasmic 100%	Cytoplasmic p-AKT with tumor grade	Nuclear p-AKT with cytoplasmic p-AKT	—
Wang and Kang [16] (48 patients: 16 grade II, 23 III, and 9 IV)	(i) Mouse monoclonal anti-AKT2 ab SCB, diluted 1 : 100 for IHC and 1 : 500 for WB (ii) p-AKT (information not provided) diluted 1 : 500 for WB	64.6% (31/48) showed cytoplasmic AKT2 staining	AKT2 and p-AKT with tumor grade	AKT2 with Ki-67	—
Suzuki et al. [17] (64 patients grade IV)	Rabbit polyclonal anti-p-AKT (Ser473) ab (CST), diluted 1 : 200 (IHC)	(i) 68.8% (44/64) (ii) 29.7% (19/64) had greater than 50% p-AKT positivity	—	—	(i) p-AKT positive, lower survival rate than p-AKT negative (ii) Multivariate analysis: higher expression of p-AKT with poor prognosis
Annovazzi et al. [18] (54 patients: 10 grade II, 10 III, and 34 IV)	(i) Mouse monoclonal anti-p-AKT (Ser473) ab (CST), diluted 1 : 100 (IHC) (ii) Rabbit monoclonal anti-p-AKT (Ser473) ab (CST), diluted 1 : 1000 (WB)	(i) 0%, 50%, and 56.6% in grade II, III, and IV, respectively (ii) Nuclear in grade II and III but mainly cytoplasmic in grade IV	p-AKT with tumor grade	p-AKT with (i) EGFR amplification (ii) p-mTOR	No significant correlation
Matsutani et al. [19] (24 patients)	Mouse monoclonal anti-AKT1 (B-1) ab (SCB) (IHC)	58.3% (14/24), cytoplasmic staining	—	(i)Positive AKT with tumor recurrences (ii) Overexpressed AKT with invasive recurrence into surrounding brain	AKT overexpression with: (i) shorter OS (ii) PFS Multivariate analysis: AKT overexpression as a significant prognostic factor for shorter PFS
Hlobilkova et al. [20] (89 patients: 42 grade I-II and 47 grade III-IV)	Mouse monoclonal anti-p-AKT (Ser473) ab (IHC)	86% of low grade and in 79% of high grade	No correlation with grade	p-AKT with EGFR activation	—
Mizoguchi et al. [21] (82 patients: 27 grade III and 55 IV)	Rabbit polyclonal anti-p-AKT (Ser473) ab (CST), diluted 1 : 100 (IHC)	(i) 78.2% of glioblastomas (43/55) positive nuclear and/or cytoplasmic (ii) 18.5% of anaplastic astrocytomas (5/27) positive nuclear and/or cytoplasmic	p-AKT with tumor grade	p-AKT with (i) EGFRvIII (ii) EGFRwt (iii) p-STAT3	p-AKT marginally predictive of worse prognosis
Pelloski et al. [22] (268 grade IV)	Rabbit polyclonal anti-p-AKT (Ser473) ab (CST), diluted 1 : 300 (IHC)	Not provided	—	p-AKT with (i) p-ERK (ii) p-p70S6K (iii) p-mTOR (iv) YKL-40	No significant correlation
Riemenschneider et al. [23] (29 grade IV)	Rabbit monoclonal anti-p-AKT (Ser473) ab (CST), diluted 1 : 50 (IHC)	Not provided	—	p-AKT with (i) p-TSC2 (ii) p-S6K (iii) p-S6	—
Wang et al. [24] (128 patients: 9 grade II, 49 III, and 70 IV)	Rabbit polyclonal anti-p-AKT (Ser473) ab (CST), diluted 1 : 50 (IHC)	p-AKT in: (i) 84% (59/70) grade IV (ii) 44% (20/46) grade III (iii) 22% (2/9) grade II	p-AKT with tumor grade	p-AKT with activated NF*κ*B	—
Chakravarti et al. [25] (11 grade II, 13 III, and 56 IV)	p-AKT (Thr308) ab (CST) (WB)	66% (50/92) of grade IV	p-AKT with tumor grade	p-AKT with (i) p-PI3K (ii) p-p70S6K (iii) inversely with cCas3	p-AKT with (i) adverse outcome (ii) reduced time to death
Choe et al. [26] (45 grade IV)	Rabbit polyclonal anti-p-AKT (Ser473) ab (CST), diluted 1 : 50 (IHC)	Not provided	—	p-AKT with (i) PTEN protein loss (ii) p-FKHR (iii) p-S6 (iv) p-mTOR	—

ab: antibody, cCas3: cleaved caspase 3, CST: Cell Signaling Technology (Beverly, MA), EP: Epitomics (CA, USA), IHC: immunohistochemistry, KPS: Karnofsky Performance Status, MVD: microvessel density, OS: overall survival, p-ERK: phosphorylated extracellular-signal-regulated kinase, PFS: progression-free survival, SCB: Santa Cruz Biotechnology, VEGF: vascular endothelial growth factor, WB: Western blot.

**Table 2:** Summary of reports investigating the role of mTOR activation in astrocytomas.

Report (number of cases and grades)	Antibody used	Immunostaining percentage	Correlations with clinicopathological features	Other correlations	Survival analysis
Korkolopoulou et al. [30] (111 patients: 25 grade II, 15 III, and 71 IV)	Rabbit monoclonal anti-p-mTOR (Ser2448) ab (CST), diluted 1 : 50 for IHC and 1 : 1.000 (WB)	(i) 84.7% (94/111) cytoplasmic/membranous (ii) 2.7% (3/111) nuclear	Gade III and IV marginally higher p-mTOR expression than grade II	p-mTOR with (i) nuclear and cytoplasmic p-AKT (ii) p-p70S6K (iii) p-4E-BP1 (iv) VEGF	p-mTOR with worse DFS
Li et al. [14] (87 patients: 27 grade I-II, 24 III, and 36 IV)	Rabbit monoclonal anti-p-mTOR (Ser2448) ab (EP), diluted 1 : 100 (IHC)	(i) 74.7% (65/87) nuclear and/or cytoplasmic (ii) 44.4% of grade IV strong expression	p-mTOR with tumor grade	—	—
Annovazzi et al. [18] (54 patients: 10 grade II, 10 III, and 34 IV)	Rabbit polyclonal anti-p-mTOR (Ser2448) ab (CST), diluted 1 : 75 (IHC)	0%, 70%, and 81.8% nuclear in grade II, III, and IV, respectively	p-mTOR with tumor grade	p-mTOR with (i) p-AKT (ii) inversely Beclin 1	No significant correlation
Pelloski et al. [22] (268 grade IV)	Anti-p-mTOR (Ser2448) ab (CST), diluted 1 : 100 (IHC)	Not provided	—	p-mTOR with (i) p-ERK (ii) p-p70S6K (iii) p-AKT (iv) PTEN (v) YKL-40	(i) p-mTOR with shorter OS (ii) Multivariate analysis: not retained as an independent prognostic factor
Riemenschneider et al. [23] (29 grade IV)	Rabbit polyclonal anti-p-mTOR (Ser2448) ab (CST), diluted 1 : 75 (IHC)	Not provided	—	No significant correlation	—
Choe et al. [26] (45 grade IV)	p-mTOR (Ser2481) ab (CST), diluted 1 : 50 (IHC)	Not provided	—	p-mTOR with (i) p-AKT (ii) p-S6 (iii) EGFRvIII	—

ab: antibody, CST: Cell Signaling Technology (Beverly, MA), DFS: disease-free survival, EP: Epitomics (CA, USA), IHC: immunohistochemistry, OS: overall survival, p-ERK: phosphorylated extracellular-signal-regulated kinase, VEGF: vascular endothelial growth factor, WB: Western blot.

**Table 3:** Summary of reports investigating the role of p70S6K and S6 activation in astrocytomas.

Report (number of cases and grades)	Antibody used	Immunostaining percentage	Correlations with clinicopathological features	Other correlations	Survival analysis
Korkolopoulou et al. [30] (111 patients: 25 grade II, 15 III, and 71 IV)	Rabbit polyclonal anti-p-p70S6K (Thr421/Ser424) ab (specific for p70 subunit) (SCB), diluted 1 : 250 for IHC and 1 : 200 for WB	99.1% (99/111) showed nuclear staining	No significant correlation	p-p70S6K with (i) cytoplasmic p-AKT (ii) p-mTOR (marginal) (iii) VEGF	No significant correlation
Yang et al. [12] (96 patients: 16 grade II, 35 III, and 45 IV)	Rabbit polyclonal anti-p-S6 (Ser235/236) ab (CST), at a concentration of 0.125 Ig/mL (IHC)	82.3% (79/96) cytoplasmic	p-S6 with (i) tumor grade (ii) lower KPS score	—	(i) p-S6 with worse prognosis (ii) Multivariate analysis: p-S6 as an independent prognostic factor
Li et al. [14] (87 patients: 27 grade I-II, 24 III, and 36 IV)	Rabbit monoclonal anti-p-p70S6K (Thr389) ab (EP), diluted 1 : 50 (IHC)	(i) 72.4% (63/87) nuclear and/or cytoplasmic (ii) 41.7% of grade IV showed strong expression	p-p70S6K with tumor grade	—	—
McBride et al. [35] (45 patients: 22 astrocytomas grade II)	(i) Rabbit polyclonal anti-p-S6 (Ser235/236) ab (SCT), diluted 1 : 200 (IHC) (ii) Rabbit polyclonal anti-p-S6 (Ser240/244) ab (CST), diluted 1 : 200 (IHC)	(i) p-S6 (Ser235/236): 76% (29/38) (ii) p-S6 (Ser240/244): 76% (29/38)	—	p-S6 (Ser235/236) with (i) p-PRAS40 (ii) p-S6 (Ser240/244) p-S6 (Ser240/244) with (iii) PTEN methylation (iv) inversely PTEN expression	(i) p-S6 (Ser235/236) with OS (ii) p-S6 (Ser240/244) with OS
Annovazzi et al. [18] (54 patients: 10 grade II, 10 III, and 34 IV)	Rabbit polyclonal anti-p-S6 (Ser240/244) ab (CST), diluted 1 : 100 for IHC. Not provided for WB	(i) 0%, 30%, and 82.3% in grade II, III, and IV, respectively (ii) Nuclear in grade II and III but mainly cytoplasmic in grade IV	p-S6 with tumor grade	p-S6 IHC with (i) p-S6 WB (ii) Ki-67 (iii) inversely with Beclin 1	No significant correlation
Ermoian et al. [36] (71 patients: 28 grade II, 17 III, and 26 IV	Anti-p-S6 ab (CST)	Not provided	p-S6 unrelated to tumor grade	No significant correlation	No significant correlation
Pelloski et al. [22] (268 grade IV)	Anti p-p70S6K ab (CST), diluted 1 : 1000 (WB)	Not provided	—	p-p70S6K with (i) p-ERK (ii) p-mTOR (iii) p-AKT (iv) PTEN (v) YKL-40	(i) p-p70S6K with shorter OS (ii) Multivariate analysis: not retained as an independent prognostic factor
Riemenschneider et al. [23] (29 grade IV)	(i) Mouse monoclonal anti-p-p70S6K (Thr389) ab (CST), diluted 1 : 200 (IHC) (ii)Rabbit monoclonal anti-p-S6 (Ser235/236) ab (CST), diluted 1 : 200 (IHC)	Not provided	—	(i) p-p70S6K with p-AKT (ii) p-S6 with p-AKT	—
Ckakravarti et al. [25] (11 grade II, 13 III, and 56 IV)	Anti-p-p70S6K (Thr389) ab (CST), diluted 1 : 50 (WB)	39.1% (36/92) of grade IV	p-p70S6K with tumor grade	p-p70S6K with (i) p-PI3K (ii) p-AKT (iii) inversely cCas3	p-p70S6K with (i) adverse outcome (ii) reduced time to death Multivariate analysis: p-p70S6K as an independent prognostic factor
Choe et al. [26] (45 grade IV)	Anti p-S6 (Ser235/236) ab (CST), diluted 1 : 50 (IHC)	Not provided	—	p- S6 with (i) EGFRwt (ii) EGFRvIII (iii) p-mTOR (iv) p-ERK	—

ab: antibody, cCas3: cleaved caspase 3, CST: Cell Signaling Technology (Beverly, MA), EP: Epitomics (CA, USA), IHC: immunohistochemistry, KPS: Karnofsky Performance Status, OS: overall survival, p-ERK: phosphorylated extracellular-signal-regulated kinase, SCB: Santa Cruz Biotechnology, VEGF: vascular endothelial growth factor, WB: Western blot.

**Table 4:** Summary of reports investigating the role of phosphorylated 4E-BP1 in astrocytomas.

Report (number of cases and grades)	Antibody used	Immunostaining percentage	Correlations with clinicopathological features	Other correlations	Survival analysis
Korkolopoulou et al. [30] (111 patients: 25 grade II, 15 III, and 71 IV)	Rabbit polyclonal anti-p-4E-BP1/2/3 (Ser36) ab (SCB), diluted 1 : 50 for IHC and 1 : 200 for WB	82.4% (61/74) nuclear	p-4E-BP1 with tumor grade	p-4E-BP1 with (i) nuclear p-AKT (ii) cytoplasmic p-ERK (iii) p-mTOR (iv) VEGF	(i) p-4E-BP1 adversely affected survival in the entire cohort and marginally in glioblastomas (ii) Multivariate analysis: p-4E-BP1 as an independent predictor of survival in the entire cohort as well as in glioblastomas
Ermoian et al. [36] (71 patients: 28 grade II, 17 III, and 26 IV	Anti-p-4E-BP1 ab (CST) (WB)	Not provided	p-4E-BP1 unrelated to tumor grade	p-4E-BP1 with p-AKT	No significant correlation
Riemenschneider et al. [23] (29 grade IV)	Rabbit polyclonal anti-p-4E-BP1 (Ser65) ab (CST), diluted 1 : 50 (IHC)	Not provided	—	No significant correlation	—

ab: antibody, CST: Cell Signaling Technology (Beverly, MA), IHC: immunohistochemistry, p-ERK: phosphorylated extracellular-signal-regulated kinase, SCB: Santa Cruz Biotechnology, VEGF: vascular endothelial growth factor, WB: Western blot.
